# A Bayesian micro-simulation to evaluate the cost-effectiveness of interventions for mastitis control during the dry period in UK dairy herds

**DOI:** 10.1016/j.prevetmed.2016.09.012

**Published:** 2016-10-01

**Authors:** P.M. Down, A.J. Bradley, J.E. Breen, W.J. Browne, T. Kypraios, M.J. Green

**Affiliations:** a University of Nottingham, School of Veterinary Medicine and Science, Sutton Bonington Campus, Loughborough LE12 5RD, United Kingdom; b Quality Milk Management Services Ltd, Cedar Barn, Easton Hill, Easton, Wells BA5 1DU, United Kingdom; c Graduate School of Education and Centre for Multilevel modelling, University of Bristol, 35 Berkeley Square, Bristol BS8 1JA, United Kingdom; d University of Nottingham, School of Mathematical Sciences, University Park, Nottingham NG7 2RD, United Kingdom

**Keywords:** Dairy cow, Mastitis control, Bayesian, Cost-effectiveness, Decision making, Dry-period

## Abstract

Importance of the dry period with respect to mastitis control is now well established although the precise interventions that reduce the risk of acquiring intramammary infections during this time are not clearly understood. There are very few intervention studies that have measured the clinical efficacy of specific mastitis interventions within a cost-effectiveness framework so there remains a large degree of uncertainty about the impact of a specific intervention and its costeffectiveness. The aim of this study was to use a Bayesian framework to investigate the cost-effectiveness of mastitis controls during the dry period. Data were assimilated from 77 UK dairy farms that participated in a British national mastitis control programme during 2009–2012 in which the majority of intramammary infections were acquired during the dry period. The data consisted of clinical mastitis (CM) and somatic cell count (SCC) records, herd management practices and details of interventions that were implemented by the farmer as part of the control plan. The outcomes used to measure the effectiveness of the interventions were i) changes in the incidence rate of clinical mastitis during the first 30 days after calving and ii) the rate at which cows gained new infections during the dry period (measured by SCC changes across the dry period from <200,000 cells/ml to >200,000 cells/ml). A Bayesian one-step microsimulation model was constructed such that posterior predictions from the model incorporated uncertainty in all parameters. The incremental net benefit was calculated across 10,000 Markov chain Monte Carlo iterations, to estimate the cost-benefit (and associated uncertainty) of each mastitis intervention. Interventions identified as being cost-effective in most circumstances included selecting dry-cow therapy at the cow level, dry-cow rations formulated by a qualified nutritionist, use of individual calving pens, first milking cows within 24 h of calving and spreading bedding evenly in dry-cow yards. The results of this study highlighted the efficacy of specific mastitis interventions in UK conditions which, when incorporated into a costeffectiveness framework, can be used to optimize decision making in mastitis control. This intervention study provides an example of how an intuitive and clinically useful Bayesian approach can be used to form the basis of an on-farm decision support tool.

## 1 Introduction

Mastitis remains one of the most costly endemic diseases to the dairy industry worldwide in terms of production, animal welfare and potential risks to public health (Bradley, 2002, Hagnestam-Nielsen and Ostergaard, 2009, Halasa, 2012). The importance of the dry period with respect to mastitis control is now well established (Bradley and Green, 2000, Bradley and Green, 2004, Green et al., 2002, Bradley et al., 2015) however the precise interventions that reduce the risk of acquiring intramammary infections (IMI) during this time are not clearly understood.

There exists a vast body of literature reporting associations between various management practices and different measures of udder health e.g. Dufour et al. (2011). A potential limitation with risk factor studies is that they cannot always provide evidence of causation and so there remains a large degree of uncertainty as to the likely impact that a specific intervention has and therefore its overall cost-effectiveness. Intervention studies can provide evidence of causation (Rubin, 2007, Martin, 2013), but there are very few intervention studies that have sought to measure the efficacy of specific mastitis control interventions within a cost-effectiveness framework (Green et al., 2010). Furthermore, uncertainty about the clinical and financial benefit of an intervention, will affect the decision to implement it (Green et al., 2010, Huijps et al., 2010). If potential interventions are to be prioritised in a rational and evidence-based way, cost-benefit analyses are required that capture the uncertainty of the efficacy of interventions.

With limited resources available to a commercial dairy farm, it is important that potential mastitis interventions are prioritised not only according to their efficacy, but also on the likely return on investment. The efficient use of available resources requires an understanding of the opportunity costs whereby resources are allocated to fund one intervention at the expense of the potential ‘benefits’ afforded by an alternative intervention. It is necessary when deciding whether to employ resources in one area to be able to compare the probability of a net benefit in that area with all other potential areas where those resources could be employed (Briggs and Gray, 1999). This is the dilemma faced by veterinary decision makers, and with many possible mastitis interventions making claims on farm resources, this decision is rarely intuitive.

Bayesian methods are now widely adopted by the human medical field for the analysis of cost-effectiveness (Briggs, 2001, Claxton et al., 2002, O’Hagan and Stevens, 2002, Spiegelhalter and Best, 2003, Claxton, 2008). A key feature of such cost-effectiveness studies is that Bayesian analysis of decision making under uncertainty requires model parameters to be specified as probability distributions (Felli and Hazen, 1999). The probability distributions represent the degree of uncertainty surrounding the true values of model parameters, which is then propagated through the model (O’Hagan, 2003). This approach makes it possible to make genuine probability statements about the magnitude of such parameters and attach a probability to a specific hypothesis of interest (Gurrin et al., 2000). One method termed comprehensive decision modelling, integrates evidence synthesis and parameter estimation with probabilistic decision analysis in a single unified framework (Cooper et al., 2004, Spiegelhalter et al., 2004, Ades et al., 2006). The resulting posterior probability distribution provides a realistic probabilistic interpretation from which statements about the probability of a hypothesis can be made.

The aim of this study was to investigate the cost-effectiveness of mastitis control interventions to reduce IMI caused by environmental pathogens (opportunistic invaders from an environmental reservoir) during the dry period. An integrated Bayesian cost-effectiveness framework was used to construct a probabilistic decision model that could be used to inform clinical decision making.

## 2 Materials and methods

### 2.1 Background to the AHDB Dairy Mastitis Control Plan (DMCP)

The DMCP (Green et al., 2007a) was delivered by local veterinary practitioners and dairy farm consultants throughout the UK who received training by the plan support team consisting of specialist dairy veterinarians. When each dairy farm was enrolled onto the DMCP, a copy of their herd health and performance data, including clinical mastitis (CM) incidence and somatic cell count (SCC) records, was submitted to the DMCP support team for analysis. The farm was subsequently visited by the plan deliverer who completed a questionnaire consisting of 377 questions covering all aspects of management relevant to mastitis control and also included observations and measurements to be collected during the visit. Each of the 377 questions/observations was associated with a corresponding intervention e.g. Question: How often are straw yards completely cleaned out? Intervention: Straw yards must be completely mucked out, at least, every 4 weeks. The answers to this questionnaire were recorded in a bespoke software programme called the ‘ePlan’ (‘SUM-IT Computer Systems Ltd,’ 2009). The DMCP support team analysed the clinical and subclinical mastitis data submitted by the plan deliverer with the aim of identifying the primary herd infection pattern i.e. predominantly environmental or contagious, and whether IMI’s were likely to be predominantly acquired during the dry period or lactating period, as described previously (Bradley et al., 2008). Each herd was assigned one of four ‘diagnoses’; environmental dry period (EDP), environmental lactation (EL), contagious dry period (CDP) or contagious lactation (CL) and this was recorded in the ePlan software. Once all of the ePlan questions and herd ‘diagnosis’ had been completed, the ePlan software was used to highlight areas of suboptimal management most relevant to the ‘diagnosis’ (Green et al., 2007a). The plan deliverer ‘prioritised’ 5–10 of these highlighted interventions for discussion with the farmer and sought agreement on which of them to implement. The mastitis performance was monitored on a regular basis thereafter by evaluating both clinical and subclinical mastitis data and a full analysis completed after a 12 month period.

### 2.2 Data collection

There were 265 plan deliverers at the time of the study and each was asked to submit their ePlan data, which consisted of the answers to the questionnaire, the interventions prioritised and the herd ‘diagnosis’ for each of the farms they had visited. They were also asked to submit the herd health and performance data recorded on-farm, consisting of CM and SCC records, herd size and milk production data, covering the 12 months prior to the DMCP start date and the first 12 months from after the plan was implemented. Out of the 265 plan deliverers, 87 plan deliverers responded. From the 87 plan deliverers that responded, ePlan data were received for a total of 452 herds that had participated in the DMCP during 2009–2012. Complete herd health and performance data were available for 290 of the 452 herds submitted. The 87 plan deliverers that had responded were asked to specify the interventions that were actually implemented on-farm over the 12 months after the initial herd visit. The plan deliverers submitted this information for 212 out of the 290 herds for which complete data were available. From the 212 herds with complete data, 77 herds were assigned an ‘EDP’ diagnosis and therefore used in this study. All of this information was collated in a Microsoft Access database (Microsoft Corp., Redmond, WA).

### 2.3 Data analysis

The clinical and subclinical mastitis data for each of the 77 herds were initially checked for completeness and any herds with incomplete records were excluded from the analysis; 73 herds out of the 77 had complete SCC data and were used for the SCC analysis and 64 herds out of the 77 had complete CM data and were therefore used for the CM analysis. In total, data from all 77 herds was used as each of those herds had at least one of complete SCC and CM data.

The outcome of interest in this research was mastitis originating from infections acquired during the dry period as reflected by clinical mastitis and somatic cell count records. Therefore to measure this, the incidence rate of clinical mastitis during the first 30 days after calving (IRCM30) was used (reported by DMCP participants as the number of cases/12 cows/month) which has been shown to be correlated to intramammary infections acquired during the dry period (Bradley and Green, 2000; Green et al., 2002), and the monthly percentage of cows that had a SCC <200,000 cells/ml at the milk recording prior to drying off, that were >200,000 cells/ml at the first milk recording after parturition (HighSCC), which has also been shown to be indicative of new dry period intramammary infections (Bradley et al., 2002, Cook et al., 2002, Bradley and Green, 2005).

Interventions that had been implemented on at least two farms were identified and for each farm, categorised as 0 (not already implemented at the time of the initial farm visit and not implemented following the intervention visit), 1 (not already implemented at the time of the initial farm visit but implemented following the DMCP) or 2 (already implemented at the time of the initial farm visit or not applicable). Interventions were classified as not applicable when they concerned an area of management not relevant to a particular farm (e.g. management of dry cow cubicles on a farm that used straw yards to house the dry cows). Collinearity between covariates was assessed using Pearson product-moment correlation coefficients, and no substantial collinearity was found (all correlation coefficients were <0.7).

**Fig. 1:**
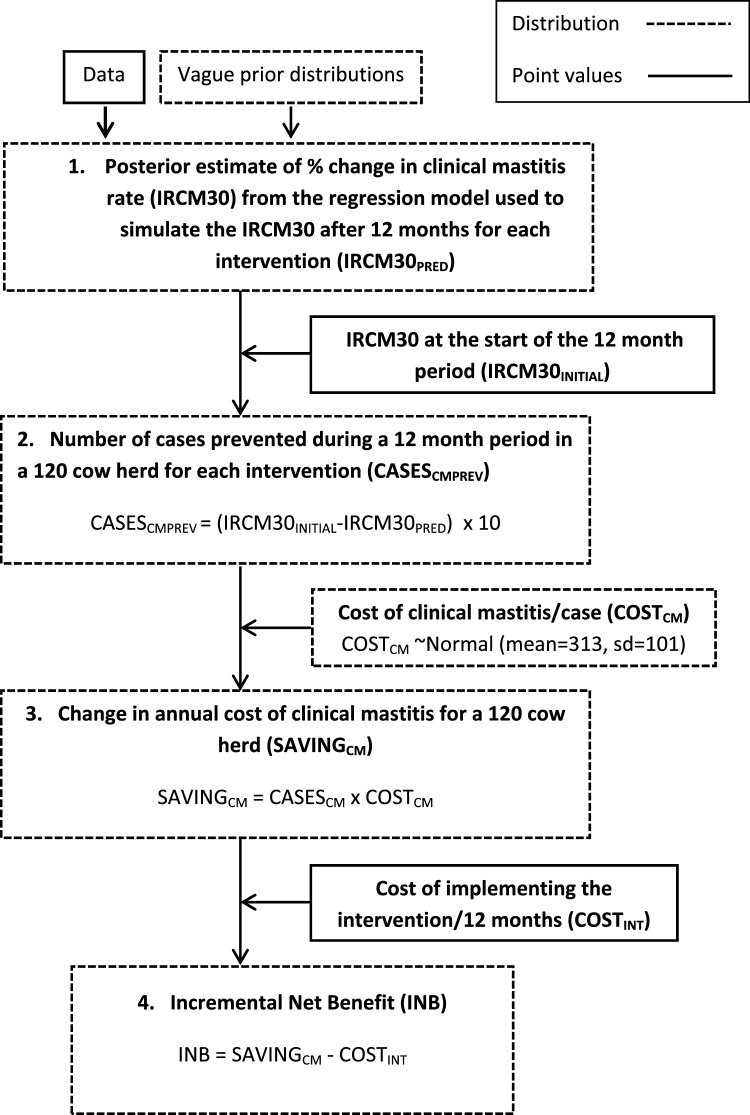
Overview of the 1-step micro-simulation procedure using the clinical mastitis micro-simulation model as an example. IRCM30 = incidence rate of clinical mastitis in the first 30 days after calving.

A Bayesian one-step micro-simulation model was constructed in OpenBUGS version 3.2.2 (Lunn et al., 2009) separately for each of the two outcomes, incorporating a multiple regression model and an onwards cost-effectiveness micro-simulation, based on methods described previously (Spiegelhalter et al., 2004). Therefore, the posterior distributions from the one-step micro-simulation model incorporated uncertainty in all model parameters (Fig. 1.).

The regression models that were incorporated in the first stage of the micro-simulation models took the form;

(1) $$Y_{i}=\beta_{0}+\beta_{1}x_{1i}+\beta_{2}x_{2i}+\beta_{3}x_{3i}+\ldots +\beta_{p}x_{pi}+\varepsilon_{i}i=1,\ldots ,n\;\varepsilon_{i}∼N\left(0,\sigma_{\varepsilon }^{2}\right)$$

where *Y*_i_ = the *i*th observation of the outcome variable, *β*_0 _= intercept value, *x*_pi_ = the *p*th predictor variable for the *i*th herd, *β_p_* = the *p*th regression coefficient, *ε*_i_ = the residual error, *p* = number of predictor variables and *n* = the number of herds.

The outcome variable (*Y*_i_) used for the clinical mastitis regression model was the percentage change in the IRCM30 during the 12 month period from implementation of the recommended interventions and the outcome variable (*Y*_i_) used in the somatic cell count regression model was the percentage change in the HighSCC rate during this 12 month period. Both of these variables were approximately normally distributed as determined using Q–Q plots. The influence of any outlying residuals was assessed using the Cook’s D value.

$$\text{Clinical mastitis regression model outcome}=\frac{\text{IRCM}30\left(12\text{ months}\right)-\text{IRCM}30\left(\text{initial}\right)}{\text{IRCM}30\left(\text{initial}\right)}\times 100$$

where IRCM30(12 months) = the mean IRCM30 during the first 12 months after the mastitis control plan started and IRCM30(initial) = the mean IRCM30 during the 12 months before the mastitis control plan started.

$$\text{Somatic cell count regression model outcome}=\frac{\text{HighSCC}\left(12\text{ months}\right)-\text{HighSCC}\left(\text{initial}\right)}{\text{HighSCC}\left(\text{initial}\right)}\times 100$$

where HighSCC(12 months) = the mean HighSCC during the first 12 months after the mastitis control plan started and HighSCC(initial) = the mean HighSCC during the 12 months before the mastitis control plan started.

Vague prior distributions were used for model parameters as follows; $\sigma_{\epsilon }^{2}$ ∼ Gamma(0.001,0.001), and β ∼ Normal(0,10^6^). The predicted values from the model for the outcome variables for each herd were compared with the observed data and displayed graphically to illustrate model performance. Full probability distributions of the intervention efficacy estimates from the regression models were carried forward into the next stages of the micro-simulation model.

The purpose of the micro-simulation was to simulate the cost-effectiveness of each intervention in theoretical herds with a herd size of 120 cows, with different initial rates of IRCM30 and HighSCC and different costs associated with implementing each intervention (Fig. 1). The values for IRCM30 and HighSCC on the simulated farms prior to interventions being implemented were taken from actual data from 125 herds that had previously participated in the DMCP so that a range of plausible scenarios were used. The micro-simulation comprised the steps described below; each step was undertaken at each model iteration.

Step 1. The regression model (1) was used to obtain an estimate of the percentage change in the IRCM30 after a 12 month period for each intervention for a given herd. The initial IRCM30 increased or decreased according to the estimated percentage change and this resulted in a predicted new IRCM30 for each farm once it had implemented the intervention (IRCM30_PRED_).

Step 2. The resulting increase/decrease in the number of cases during a 12 month period (CASES_CMPREV_) in a 120-cow herd was then simulated for each intervention individually by multiplying the difference between the initial IRCM30 (IRCM30_INITIAL_) and the predicted IRCM30 (IRCM30_PRED_) by 10 to convert the denominator from the number of cases per 12 cows, to the number of cases per 120 cows:

$$CASES_{CMPREV}=(IRCM30_{INITIAL}-IRCM30_{PRED})\times 10$$

Step 3. The change in annual cost of clinical mastitis for a 120 cow herd (SAVING_CM_) was calculated at each iteration by multiplying the number of cases prevented (CASES_CMPREV_) by the cost of a case of clinical mastitis (COST_CM_):

$$SAVING_{CM}=CASES_{CM}\times COST_{CM}$$

A cost per case of clinical mastitis within 30 days of calving was specified as a full probability distribution, COST_CM_ ∼ Normal (mean = 313, sd = 101), based on a stochastic simulation study in the UK (Green et al., 2009). A cost was selected at random from this distribution at each iteration and multiplied by the number of cases prevented to give an overall saving in pounds sterling associated with the implementation of each intervention.

Step 4. The incremental net benefit (INB) was calculated at each iteration to represent the overall net benefit after all ‘savings’ and ‘costs’ had been considered over the 12 month period:

$$INB_{CM}=SAVING_{CM}-COST_{INT}$$

The cost of implementing each intervention (COST_INT_) was specified as one of four different values taken from across a plausible spectrum ranging from a ‘low cost’ scenario (£250/12 months) to a ‘high cost’ scenario (£1000/12 months). Due to the huge inter-farm variation in the cost of implementing mastitis interventions, any specified range could be considered to be arbitrary. Therefore, rather than trying to predict the actual cost of implementing specific interventions, a range of values was specified to provide an indication as to how much ‘room for investment’ there was for each specific intervention. The actual cost of implementation can be entered in the decision support tool in order to make farm-specific predictions.

Parameters throughout the model were estimated from 10,000 Markov chain Monte Carlo (MCMC) iterations, following a burn-in of 1000 simulations. Three chains starting at ‘overdispersed’ initial values were simulated and convergence was assessed by comparing intra- and inter-chain variability using the Brooks-Gelman-Rubin diagnostic (Brooks and Gelman, 1998, Gelman and Rubin, 1992).

**Fig. 2:**
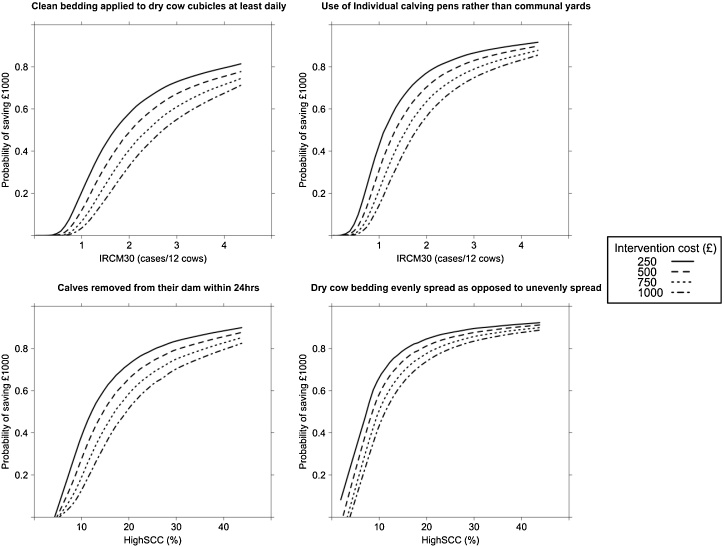
Probabilistic cost-effectiveness curves for specific interventions. IRCM30 = the incidence rate of clinical mastitis in the first 30 days after calving. HighSCC = monthly percentage of cows that had a somatic cell count <200,000 cells/ml at the milk recording prior to drying off, that were >200,000 cells/ml at the first milk recording after parturition.

An indicator variable was set to 1 at each intervention when the micro-simulation model predicted an INB of £1000 or greater and otherwise to 0. The mean value of this indicator over the 10,000 iterations provided an estimate of the probability of exceeding a return of £1000. Predictions of INB were plotted for each of the four different values of COST_INT_ to produce probabilistic cost-effectiveness curves that display the probability of saving, at least, £1000 over 12 months at different levels of mastitis for each intervention (Fig. 2). A cut point probability of ≥60% for a saving ≥£1000 in a 12 month period was used to label interventions as potentially cost-effective; these interventions are reported. A saving of £1000 in a 12 month period was considered by the authors to be a worthwhile saving for demonstration purposes but farmers will be able to stipulate their own desired level of saving in the decision support tool. An alternative approach would have been to simply provide the resulting posterior distribution for the INB, however, by selecting the threshold of £1000, we were able to demonstrate how the posterior distribution can be used to provide intuitive predictions for clinical decision makers.

### 2.4 Somatic cell count micro-simulation model

The micro-simulation steps took the same form for the somatic cell count micro-simulation model except the cost of a case of HighSCC was defined by the normal distribution; COST_SCC_ ∼ Normal (mean = 290, sd = 112) (Green et al., 2009).

## 3 Results

### 3.1 Herd parameters

The median size of the 77 herds selected for analysis was 187 cows (range 51–553) and the median 305d milk yield was 8611 kg (range 4297–10590). The median incidence rate of CM in the 12 months prior to mastitis interventions was 59.5 cases/100 cows/year (range 18–164) and the median 12-month average BMSCC was 206,000 cells/ml (range 74,000–398,000). The median IRCM30 at the time of the initial herd visit was 13 cases/100 cows/month (12-month average, range 0.25–36.25) and the median HighSCC rate was 18.35%/month (12-month average, range 1.9–43.8).

### 3.2 Interventions

A total of 112 interventions were evaluated in the analysis and the number of farms implementing each of the interventions ranged from 2 to 15. Interventions that were found to be cost-effective in most scenarios were reported resulting in 13 interventions for the CM model and 9 interventions for the SCC model. The reported interventions could be broadly grouped into three categories; management of the dry cow environment, management of the calving cow environment and the selection and application of dry cow therapy (Table 1, Table 2).

### 3.3 Micro-simulation models

#### 3.3.1 Regression model fit

**Fig. 3:**
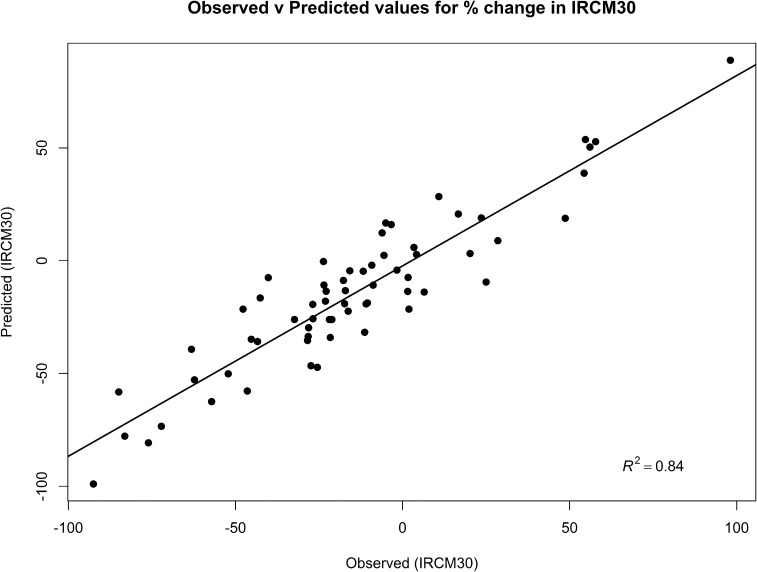
Scatterplot of observed and predicted values of the percentage change in the incidence rate of clinical mastitis in the first 30 days after calving (IRCM30) in the 12 months since the mastitis control plan was instigated.

**Fig. 4:**
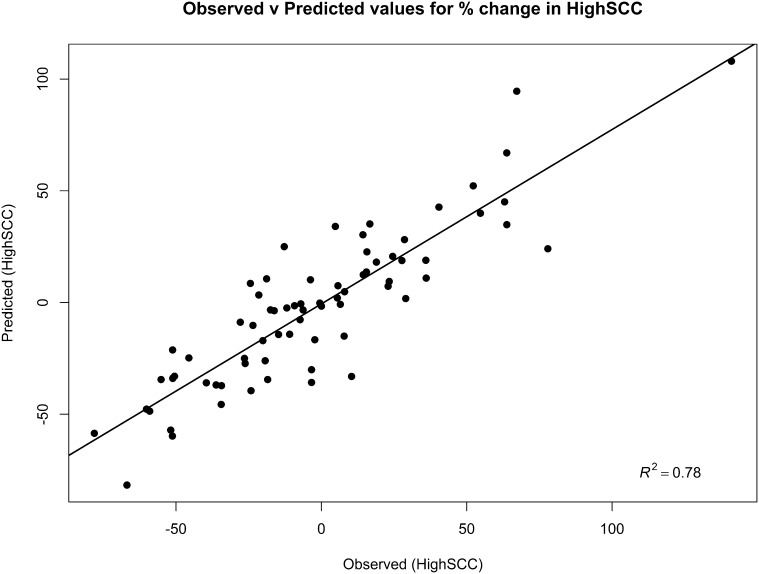
Scatterplot of the observed and predicted values of the percentage change in the monthly percentage of cows that had a somatic cell count <200,000 cells/ml at the milk recording prior to drying off, that were >200,000 cells/ml at the first milk recording after parturition (HighSCC) in the 12 months since the mastitis control plan was instigated.

Both regression models demonstrated a good ability to predict the incidence rate of IRCM30 and HighSCC for a given farm, with the model predictions explaining over 84% of the variability in the observed data in the clinical mastitis regression model (Fig. 3) and 78% in the somatic cell count regression model (Fig. 4).

#### 3.3.2 Cost-effectiveness outcome

The probability of an incremental net benefit of at least £1000 for different interventions is provided in Table 1, Table 2 and Fig. 2. Interventions in the clinical mastitis micro-simulation model that were cost-effective for most farms (>75% probability of saving £1000 with initial IRCM30 of 2 cases/12 cows and a COST_INT_ of £500) were dry cow rations being formulated by a suitably qualified nutritionist as opposed to an unqualified person, selecting dry cow therapy (DCT) at cow level (selective) rather than at herd level (blanket), balancing calcium and magnesium in the dry cow rations, designing cubicles in such a way that 90% of dry cows lied in them correctly and not drying-off cows during foot trimming procedures. The interventions in the somatic cell count micro-simulation model that were cost-effective for most farms (>75% probability of saving £1000 with initial HighSCC rate of 20% and a COST_INT_ of £500) were spreading bedding evenly in dry cow yards as opposed to poor bedding spreading, abrupt drying off as opposed to once daily milking and calving in individual pens as opposed to communal yards.

Interventions in the clinical mastitis micro-simulation model that were sensitive to the cost of the intervention and the initial IRCM30 and therefore only likely to be cost-effective in certain scenarios included cleaning dry cow cubicles at least twice daily, calving in individual calving pens as opposed to communal yards, milking cows for the first time within 24 h of calving and considering both antibiotic and non-antibiotic dry cow therapy approaches for low somatic cell count cows. Interventions in the somatic cell count micro-simulation model that were sensitive to the cost of the intervention and the initial HighSCC rate included milking cows for the first time within 24 h of calving, removing calves from the cow within 24 h of birth and differentiating infected from uninfected cows at drying off using SCC records from the current lactation.

## 4 Discussion

This study illustrates how the clinical efficacy of specific mastitis interventions can be quantified and incorporated into a Bayesian cost-effectiveness model using a one-stage micro-simulation. This is the first intervention study to explore the cost-effectiveness of mastitis interventions within a Bayesian framework, the results of which are to be incorporated into a decision support tool that will be made available to veterinarians/advisors involved with implementing the AHDB Dairy Mastitis Control Plan in the United Kingdom.

Interventions relating to the design and comfort of dry cow cubicles such as designing cubicles in such a way that cows lie in them correctly and removing dung and wet bedding from cubicles at least twice daily were potentially cost-effective interventions in the clinical mastitis micro-simulation, and aspects of management related to these have been highlighted previously (Barkema et al., 1999). This earlier study identified the type of cubicle divider and thickness of cubicle bedding to be associated with the incidence rate of clinical mastitis. The hygiene of dry cow cubicles has been associated with changes in bulk milk somatic cell count (Barkema et al., 1998) and clinical mastitis incidence rate (Schukken et al., 1991, Green et al., 2007b), and this highlights the need to provide comfortable, clean cubicles for dry cows as well as lactating cows.

Another aspect of the dry cow environment which is important but commonly overlooked is the grazing management and specifically the rotation of paddocks. In this study a ‘graze 2, rest 4’ policy was used (paddocks are grazed for no more than 2 consecutive weeks and then rested for no less than 4 weeks), and whilst the effect of this intervention was relatively small in each of the micro-simulation models, the combined predicted reduction in clinical mastitis and somatic cell count would make this an intervention likely to be cost-effective, providing the cost to implement it was modest. This is in agreement with two previous studies that found this intervention to be associated with reduced somatic cell counts and clinical mastitis incidence in UK dairy herds (Green et al., 2007b, Green et al., 2008), and emphasises the need to consider pasture contamination and ways to mitigate these risks.

The use of individual calving pens, as opposed to communal yards, had a high probability of cost-effectiveness, and this has been identified as an important risk factor by previous studies (Hutton et al., 1991, Bartlett et al., 1992, Barkema et al., 1998, Barnouin et al., 2004, O’Reilly et al., 2006). This effect may be due to a reduction in pathogen exposure but may also reflect indirectly, the negative impact of cross-suckling calves which has been associated with clinical mastitis incidence in the current study and previously (Green et al., 2007a). Cross-suckling would also be less likely to occur when calves are removed within 24 h of calving and this intervention was associated with a moderate probability of a £1000 return in the somatic cell count micro-simulation model.

Selecting dry cow therapy at cow level was associated with a reduced IRCM30, as has been reported previously (Green et al., 2007b). Since neither the products used nor the criteria applied to select between cows was specified in this study, it remains unclear whether some approaches to selective dry cow therapy are superior to others.

Having a policy of using both antibiotic and non-antibiotic approaches when drying-off low somatic cell count cows was predicted to reduce IRCM30 and was very likely to be a cost-effective intervention. Importantly, such a policy will also reduce the quantity of antimicrobial usage on farm. With the increasing concerns about antibiotic resistance comes an increasing pressure on dairy farmers to reduce antibiotic usage (Call et al., 2008, Oliver et al., 2011). In herds such as these with a low prevalence of contagious mastitis and a relatively low bulk milk somatic cell count, the targeting of antibiotic dry cow therapy at cows infected at drying off and use of non-antibiotic teat-sealants in uninfected cows is a rational and effective approach to dry cow therapy (Huxley et al., 2002, Green et al., 2008, Bradley et al., 2010, Cameron et al., 2015). Irrespective of which dry cow therapy products are used at drying-off, interventions affecting the hygiene of the procedure itself, and the cleanliness of the environment in which it is performed, were shown to be potentially cost-effective in both models and confirms previous study findings (Peeler et al., 2000, Barnouin et al., 2004, Barnouin et al., 2005, Green et al., 2008).

Two interventions in the clinical mastitis micro-simulation model that were predicted to be highly cost-effective in most scenarios were the formulation of dry cow rations by a suitably qualified nutritional adviser and the balancing of calcium and magnesium to prevent milk fever. This is also in agreement with other studies investigating the role of nutrition in mastitis control (Kremer et al., 1993, Oltenacu and Ekesbo, 1994, O’Rourke, 2009). These studies reported that mastitis was more likely to occur in cows diagnosed with clinical ketosis and cows deficient in vitamins and trace elements such as selenium, vitamin E, copper, zinc, vitamin A and β-carotene.

The uncertainty in clinical and financial outcome for an individual farm is important, and illustrates the usefulness of using a probability distribution for anticipated financial returns. The integrated Bayesian model used in this analysis simultaneously derived the joint posterior distribution for all unknown parameters and propagated the effects through the predictive cost-effectiveness model. In this example, uncertainty in the cost of mastitis for each herd is included as well as the uncertainty of the effects of the interventions. There are several advantages of this approach, which have been outlined previously (Spiegelhalter and Best, 2003, Spiegelhalter et al., 2004). The main disadvantages of the unified Bayesian approach include the need for full MCMC software in order to obtain a solution although this is currently freely available (Lunn et al., 2000) and it can also be difficult to evaluate or check model accuracy (Green et al., 2010). Such unified Bayesian models are used widely in human medicine (Parmigiani, 2002, Spiegelhalter et al., 2004), but there are few examples in the veterinary literature (Green et al., 2010, Archer et al., 2014). They provide a useful method to improve the understanding of the uncertainties involved in clinical decision making and therefore have much to offer the decision analyst and decision-maker (Cooper et al., 2004).

The results of this research were incorporated into a spreadsheet-based decision support tool to enable vets and farmers to explore different scenarios applicable to them. Farm-specific parameters can be entered and required savings specified, resulting in predictions that are relevant to each individual farm. Information regarding the herd size, current clinical and subclinical mastitis performance and costs can be inputted in addition to the cost of implementing each intervention. The level of saving required after 12 months is then specified according to the farmers needs and the decision support tool calculates the probability of making the specified level of return and displays this as a probability distribution so the uncertainty can be visualised. The decision support tool also allows different combinations of interventions to be evaluated simultaneously so that many different scenarios can be explored.

This research measured the cost-effectiveness of mastitis interventions in herds specifically with an ‘EDP’ diagnosis and as such it is difficult to know how these findings would translate to herds more generally. The results may have been influenced by participation bias due to characteristics common to the plan deliverers that submitted data compared with those that didn’t. It is also likely that the results are biased towards herds seeking veterinary input with respect to mastitis control rather than being representative of the national herd as a whole. However, the data most likely provides a true reflection of dairy herds seeking veterinary input with respect to mastitis control and is, therefore, of value to those involved in the delivery of these services.

## 5 Conclusions

In this study, data from 77 UK dairy herds were used to explore the cost-effectiveness of specific mastitis control interventions in herds with a particular problem with IMI acquired during the dry period. Results from the Bayesian micro-simulation models identified that a variety of interventions would be cost effective in different farm circumstances. The cost-effectiveness of different interventions has been incorporated in a decision support tool to assist optimal decision making by veterinary practitioners in the field.

## Conflict of interest statement

The authors have no conflicts of interest

## Figures and Tables

**Table 1:** Probability of saving at least £1000 after 12 months at different incidence rates of clinical mastitis in the first 30 days after calving (IRCM30) and for different costs of implementing the intervention. Interventions ranked from most cost-effective to least.

		Cost of Intervention (£)			
Intervention	IRCM30 (cases/12 cows)	250	500	750	1000
Dry cow rations should be formulated by a suitably qualified nutritional advisor	1.5	0.92	0.89	0.85	0.80
	2.0	0.95	0.94	0.92	0.89
	3.0	0.98	0.97	0.96	0.95
Dry cow therapy (DCT) should be selected at the cow level (a suitable product for each cow) rather than herd level	1.5	0.84	0.76	0.67	0.57
	2.0	0.92	0.88	0.83	0.77
	3.0	0.97	0.96	0.93	0.91
Calcium and magnesium should be balanced to prevent milk fever	1.5	0.77	0.71	0.65	0.58
	2.0	0.85	0.81	0.77	0.71
	3.0	0.90	0.88	0.86	0.84
Cows must not be dried off during foot-trimming	1.5	0.76	0.70	0.63	0.56
	2.0	0.83	0.79	0.75	0.70
	3.0	0.89	0.87	0.85	0.82
Cubicles should be designed such that at least 90% of dry cows will lie in them correctly at all times	1.5	0.74	0.67	0.60	0.54
	2.0	0.82	0.78	0.73	0.68
	3.0	0.88	0.86	0.84	0.81
Dung, soiling and wet bedding should be removed at least twice daily from dry cow cubicles	1.5	0.70	0.59	0.48	0.38
	2.0	0.83	0.76	0.68	0.60
	3.0	0.92	0.89	0.85	0.80
Cows should be milked for the first time within 24 h of calving	1.5	0.70	0.60	0.50	0.40
	2.0	0.81	0.75	0.68	0.60
	3.0	0.90	0.87	0.83	0.79
Cows should calve in individual pens rather than communal yards	1.5	0.65	0.56	0.47	0.39
	2.0	0.76	0.70	0.63	0.56
	3.0	0.86	0.82	0.78	0.74
Both antibiotic and non-antibiotic DCT approaches should be considered for low somatic cell count cows	1.5	0.50	0.39	0.29	0.21
	2.0	0.65	0.56	0.47	0.39
	3.0	0.80	0.74	0.68	0.62
Clean bedding material should be applied to dry cow cubicles at least once daily if using organic bedding	1.5	0.46	0.36	0.27	0.20
	2.0	0.61	0.52	0.44	0.36
	3.0	0.76	0.70	0.64	0.58
Pasture must not be grazed for more than 2 consecutive weeks and must be rested for at least 4 weeks before cows are returned to graze	1.5	0.41	0.33	0.26	0.20
	2.0	0.52	0.45	0.39	0.33
	3.0	0.63	0.59	0.54	0.49
Straw yards for calving cows should be cleaned out completely at least once per month	1.5	0.40	0.28	0.20	0.13
	2.0	0.58	0.47	0.37	0.29
	3.0	0.74	0.67	0.60	0.53
Calves must only be allowed to suckle their own dam to prevent the possible transfer of pathogens in milk between cows	1.5	0.28	0.19	0.12	0.07
	2.0	0.44	0.35	0.26	0.19
	3.0	0.64	0.56	0.48	0.41

**Table 2:** Probability of saving at least £1000 after 12 months at different monthly percentages of cows that had a SCC <200,000 cells/ml at the milk recording prior to drying off, that were >200,000 cells/ml at the first milk recording after parturition (HighSCC) and different costs of implementing the intervention. Interventions ranked from most cost-effective to least.

		Cost of Intervention (£)			
Intervention	HighSCC%	250	500	750	1000
Cows should calve in individual pens rather than yards rather than communal yards	15	0.84	0.78	0.72	0.65
	20	0.90	0.86	0.83	0.78
	30	0.95	0.93	0.91	0.89
Drying off must be abrupt; that is, cows should not be milked once daily in the days prior to drying-off	15	0.84	0.78	0.72	0.65
	20	0.90	0.86	0.83	0.78
	30	0.95	0.93	0.91	0.89
Bedding should be spread evenly rather than unevenly in straw yards for dry cows	15	0.80	0.76	0.71	0.66
	20	0.86	0.83	0.79	0.76
	30	0.90	0.89	0.87	0.85
There must be good ventilation but without draughts in all calving cow housing	15	0.64	0.56	0.48	0.41
	20	0.73	0.67	0.61	0.55
	30	0.82	0.79	0.75	0.71
The calf should be removed from the cow within 24 h of birth after ensuring colostrum has been fed	15	0.62	0.53	0.44	0.36
	20	0.74	0.66	0.60	0.52
	30	0.84	0.80	0.76	0.72
Cows should be milked for the first time within 24 h of calving	15	0.59	0.49	0.40	0.32
	20	0.72	0.64	0.56	0.48
	30	0.83	0.79	0.74	0.69
Dry cow therapy must be administered hygienically, as detailed in the standard operating procedure provided with the training materials	15	0.52	0.43	0.34	0.26
	20	0.65	0.57	0.49	0.42
	30	0.78	0.73	0.68	0.63
You should differentiate infected from uninfected cows using somatic cell count records from the current lactation	15	0.42	0.34	0.27	0.21
	20	0.54	0.47	0.40	0.34
	30	0.66	0.61	0.56	0.51
Each quarter should be stripped within 4 h of calving to check for mastitis	15	0.38	0.29	0.22	0.16
	20	0.52	0.44	0.36	0.29
	30	0.68	0.62	0.55	0.49
